# TRAIL Is Decreased Before 20 Weeks Gestation in Women with Hypertensive Disorders of Pregnancy

**DOI:** 10.1371/journal.pone.0128425

**Published:** 2015-06-01

**Authors:** Cheng Zhou, Yan Long, Hongling Yang, Chunyan Zhu, Qingling Ma, Yonggang Zhang

**Affiliations:** 1 Department of Clinical Laboratory, Guangzhou Women and Children's Medical Center, Guangzhou Medical University, Guangzhou, Guangdong, P.R. China; 2 School of Public Health, Guangzhou Medical University, Guangzhou, Guangdong, P.R. China; University of Barcelona, SPAIN

## Abstract

**Objective:**

The present study evaluated maternal plasma protein profiles before the onset of hypertensive disorders of pregnancy (HDP) to assess the relationship between maternal plasma tumor necrosis factor-related apoptosis-inducing ligand (TRAIL) and HDP before 20 weeks gestation and to evaluate the discriminatory performance of plasma TRAIL levels for HDP.

**Methods:**

A 2-phase discovery/validation study was designed. In the discovery phase, a nested case-controlled study was performed using plasma sampled at 8 to 20 weeks gestation from 20 women who later developed HDP and from 20 age- and gestational week-matched controls. Plasma was analyzed using a human protein microarray technology designed to simultaneously detect 507 proteins. The functional annotation and clustering of the differentially expressed proteins were performed using DAVID and the GO database. TRAIL levels were further validated in an independent study using plasma obtained at 8 to 20 weeks gestation from 53 women who later developed HDP and from 106 matched controls, and 62 clinical risk factors were investigated.

**Results:**

In the protein microarray analysis, 23 proteins were differentially expressed between the two groups. The ELISA showed that women who later developed HDP had significantly lower TRAIL levels compared to women with uncomplicated pregnancies. The multivariable Cox regression analysis identified the following three factors that were entered into the final Cox regression model: gravidity (OR = 2.02, 95% CI 1.00–4.09), pre-pregnancy BMI (OR = 1.46, 95% CI 1.21–1.76) and TRAIL levels (OR = 0.97, 95% CI 0.94–0.99). The model had a significantly better discriminatory power (AUC = 0.83, 95% CI 0.75–0.88) compared to TRAIL alone as an independent predictor of HDP (AUC = 0.59, 95% CI 0.51–0.67).

**Conclusion:**

Twenty-three differentially expressed proteins before 20 weeks gestation might be associated with the pathogenesis of HDP. Plasma TRAIL levels were associated with the development of HDP, and the combination of plasma TRAIL levels with pre-pregnancy BMI and gravidity had a good discriminatory performance for HDP before 20 weeks gestation.

## Introduction

Hypertensive disorders of pregnancy (HDP) is a pregnancy-specific syndrome defined clinically as hypertension with or without proteinuria after 20 weeks gestation [1]. HDP includes gestational hypertension and preeclampsia, which occurs in 3–5% of pregnancies and is the most common cause of maternal and fetal death worldwide [2]. HDP is associated with intrauterine growth restriction and prematurity [3]. Surviving neonates are at risk of developing neurodevelopmental disabilities, such as cerebral palsy and mental retardation. Furthermore, women with HDP have an increased risk of subsequently developing cardiovascular disease, diabetes mellitus, stroke and hypertension [4].

As a result, there is great interest in the early identification of women at risk for HDP to employ prevention and intervention strategies. Numerous biomarkers have been proposed for predicting HDP, including pregnancy-associated plasma protein A, placental protein 13, placental growth factor and endoglin [5],[6],[7]. However, to date, no single factor or combination of factors has exhibited adequate sensitivity and specificity for clinical use [8]. The reported HDP detection rate when screening using a combination of maternal factors is approximately 30%, with a 5% false positive rate [9].

Preeclampsia is considered to be a consequence of incomplete trophoblast invasion and spiral artery remodeling [10],[11]. This results in placental hypoxia and the release of placental factors into maternal circulation, which causes widespread endothelial damage [12]. Plasma proteins are involved in placental implantation, and these specific proteins act as placental factors that contribute to endothelial dysfunction and many other pathophysiological changes that are related to preeclampsia [13]. Plasma protein alterations precede the clinical onset of preeclampsia, supporting the idea that these proteins contribute to preeclampsia [14]. Because it is a complex, multifactorial syndrome, we used antibody microarray technology to determine the key predictive proteins associated with the syndrome.

Tumor necrosis factor-related apoptosis-inducing ligand (TRAIL) belongs to the TNF ligand family and can induce apoptosis by interacting with death receptors [15],[16]. TRAIL and its receptors are ubiquitously expressed in the placenta, where they play a vital role in trophoblastic immune privilege and trophoblast invasion during early pregnancy [17],[18],[19]. Throughout pregnancy, placental microparticles, which might contain fragments expressing TRAIL or sTRAIL, are increasingly produced and released into the maternal blood circulation [20]. Thus, there is interest in ascertaining the potential difference in plasma TRAIL concentration between those with and without HDP. This study sought to improve the discriminatory performance of HDP by combining the novel factor TRAIL with previously reported clinical risk factors.

## Materials and Methods

### Subjects

All women who had attended the antenatal clinic and subsequently delivered at Guangzhou Women and Children’s Medical Center between April 2013 and July 2014 were recruited. The study protocol was approved by the Ethics Committee of Guangzhou Women and Children’s Medical Center. Written informed consent was obtained from all participants.

HDP was defined as hypertension (≥140 mmHg systolic pressure or ≥90 mmHg diastolic pressure on two or more occasions at least 6 h apart after 20 weeks gestation in a woman with previously normal blood pressure) with or without proteinuria. On the other hand, normal control subjects exhibited normal blood pressure, without proteinuria, fetal malformations or other pregnancy complications. For both cases and controls, we excluded women with a multiple pregnancy, gynecological disease, gestational diabetes mellitus, liver disease or kidney disease.

**Fig 1:**
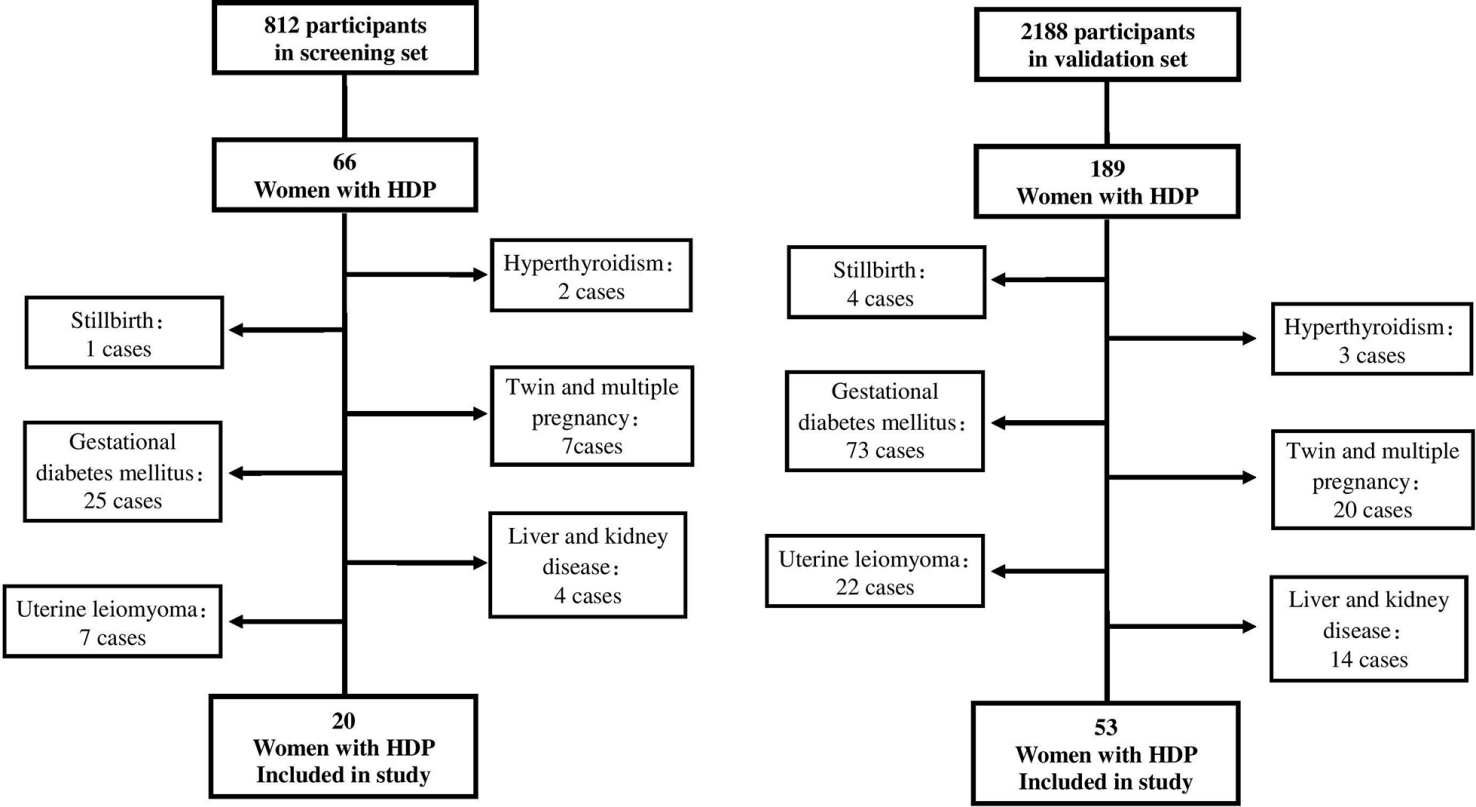
Flow diagram of participants in the screening and validation studies.

In the nested case-control study during the discovery phase, 812 women were recruited. HDP developed in 66 participants, 20 of whom satisfied all the inclusion and exclusion criteria; the remainder were excluded (Fig 1). These 20 women who developed HDP were matched in a 1:1 ratio for age, gestational week and sample date to 20 controls who experienced an uncomplicated pregnancy.

In the nested case-control study during the validation phase, 2188 women were recruited. HDP developed in 189 patients, 53 of whom satisfied all the inclusion and exclusion criteria; the remainder were excluded (Fig 1). These 53 women who developed HDP were matched in a 2:1 ratio for age, gestational week and sample date to 106 controls who experienced an uncomplicated pregnancy.

### Sample collection

Peripheral venous blood samples were taken from women at 8–20 weeks gestation and centrifuged at 3000×*g* (10 min at room temperature). Aliquots of plasma were stored immediately at -80°C.

### Risk factors study

Possible risk factors were selected based on a literature review and clinical experience. Data were obtained from medical records. The variables included sociodemographic characteristics (maternal age, pre-pregnancy BMI, and maternal smoking), pregnancy history, past medical history (gestational diabetes mellitus, HDP, liver disease and kidney disease), family history (gestational diabetes mellitus, HDP, and hypertension), conditions during pregnancy, and the husband's medical history.

### Protein microarray analysis

A RayBio Label-based Human Antibody Microarray (catalog # AAH-BLG-1; RayBiotech, Norcross, GA, USA) was used to detect 507 proteins of interest. Assays were performed in accordance with the manufacturer's instructions. Briefly, serum samples were dialyzed in a dialysis tube and were subsequently labeled with biotin. After blocking with blocking buffer, the chips were incubated with biotin-labeled plasma for 2 h at room temperature. After extensive washing to decant the unbound sample, the chips were incubated with fluorescent dye-conjugated streptavidin for 2 h at room temperature. The chips were washed and then scanned using a Genepix 4000B Microarray Scanner (Axon Instruments, Sunnyvale, CA, USA). Spots were digitized into raw signal intensities, which were imported into the RayBio Analysis Tool to subtract the local background and to normalize the signal intensities to the internal positive and negative controls prior to data analysis.

### ELISA

Due to the difficulties in obtaining early pregnancy plasma samples from women with HDP, we only validated TRAIL levels because they exhibited the highest fold-change among the down-regulated proteins in the protein microarray, and TRAIL plays an important role in trophoblastic immune privilege and trophoblast invasion during early pregnancy. Plasma concentrations of TRAIL (catalog no. DTRL00; R&D Systems, Minneapolis, MN, USA) were measured by ELISA. The intra-assay variation coefficient was 5.6%, and the inter-assay variation was 4.4%. The lowest TRAIL concentration detectable was 8.0 pg/ml. Assays were performed in accordance with the manufacturer's instructions.

### Statistical analysis

In the screening phase, the intensity value of each protein was expressed as the mean ± SD, and differences between two groups were compared using two independent t-tests. The functional annotation and clustering of the differentially expressed proteins were performed using Database for Annotation, Visualization and Integrated Discovery (DAVID) and the Gene Ontology (GO) database.

In the validation phase, data were analyzed by Cox regression (forward stepwise selection with the likelihood ratio criterion; the inclusion and exclusion criteria were *P*≤0.05 and *P*>0.10, respectively). Significant variables determined by the univariate Cox regression were entered into the multivariable Cox regression model to assess the relationship between risk factors and HDP. The goodness of fit for Cox model was assessed using the -2 log likelihood (-2Log L) statistic. Receiver operating characteristic (ROC) analysis was used to evaluate the model's discriminatory performance for HDP.

Statistical analyses were performed using SPSS 18.0 for Windows and MedCalc 11.4. *P*<0.05 was considered statistically significant.

## Results

### Detection of plasma protein by antibody microarray

**Fig 2:**
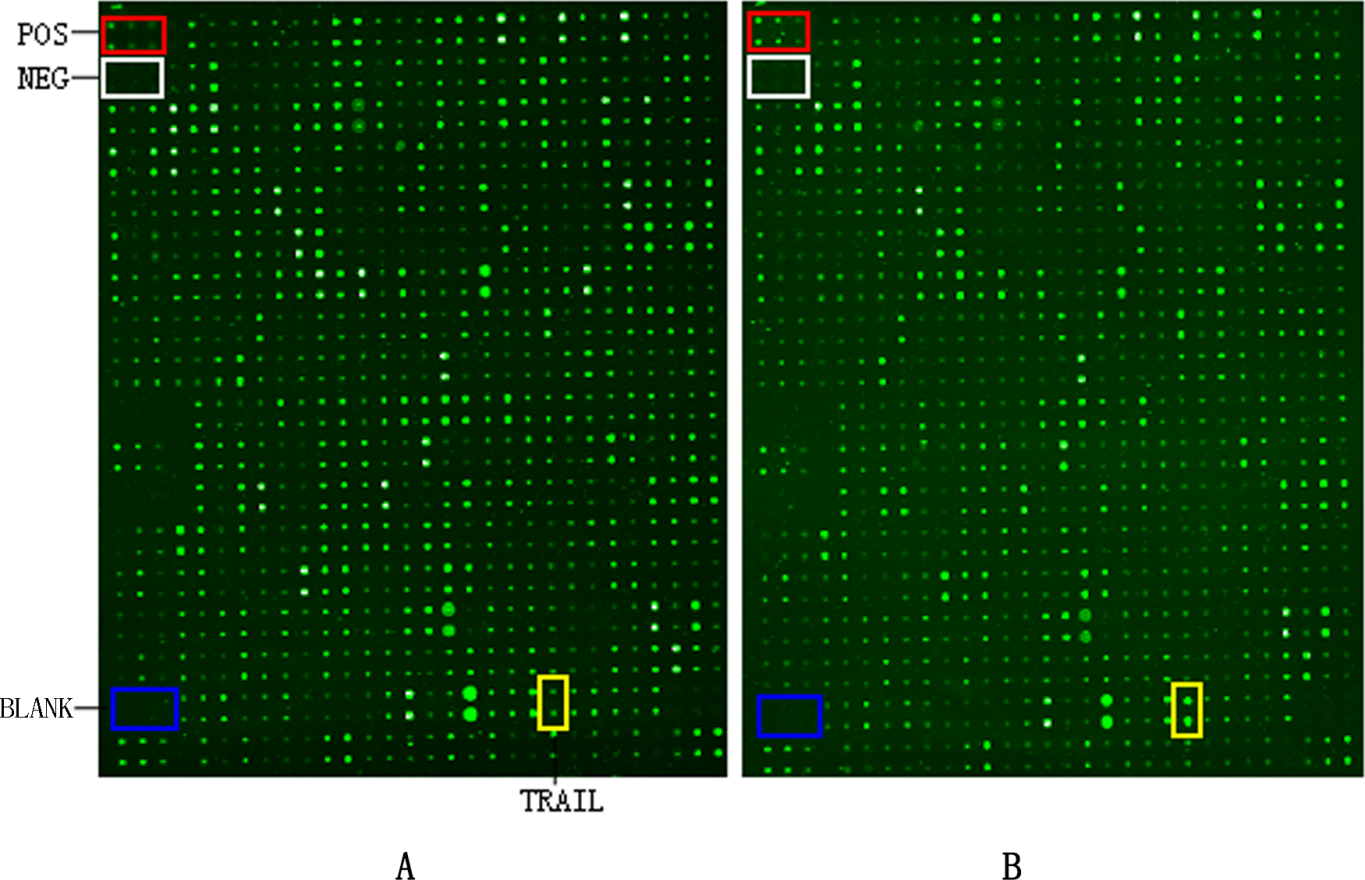
Image of the RayBio Human Antibody Microarray. A total of 507 antibodies were placed on the microarray. Representative microarray membranes incubated with plasma from 20 women who later developed HDP (A) and 20 women who experienced an uncomplicated pregnancy (B). POS: positive control; NEG: negative control; TRAIL: tumor necrosis factor-related apoptosis-inducing ligand.

Plasma samples from women in both groups were analyzed with a RayBio Human Antibody Microarray, and 507 proteins were detectable in the microarray (Fig 2). Compared to healthy pregnant controls, 23 proteins were differentially expressed in the HDP group. Of these, 12 proteins (including TRAIL and IGFBP-2) were up-regulated (Table 1) and 11 proteins were down-regulated (Table 1) in the HDP group. Impressively, TRAIL exhibited the highest fold-change (HDP/control) among the down-regulated proteins.

**Table 1:** Fold change and ROC analysis of differentially expressed proteins in pregnant women with and without hypertensive disorders of pregnancy.

Protein	Fold change (HDP/control)	*P*	AUC (95% CI)	*P* (ROC)
GITR/TNFRF18	0.87	0.044	0.77 (0.62–0.92)	0.004
IGFBP-2	0.83	0.003	0.76 (0.61–0.91)	0.005
TRAIL/TNFSF10	0.80	0.021	0.75 (0.63–0.91)	0.007
beta-NGF	0.85	0.015	0.75 (0.58–0.91)	0.007
GFR alpha-4	0.86	0.049	0.74 (0.59–0.90)	0.009
TIMP-4	1.24	0.003	0.73 (0.62–0.88)	0.009
Endoglin	1.28	0.032	0.73 (0.57–0.89)	0.012
IL-17RD	1.51	0.002	0.73 (0.57–0.90)	0.012
Dkk-1	0.83	0.017	0.73 (0.57–0.89)	0.012
CXCR3	0.80	0.025	0.73 (0.57–0.88)	0.015
Activin A	0.82	0.012	0.71 (0.54–0.87)	0.027
Eotaxin/CCL11	0.84	0.046	0.70 (0.54–0.86)	0.030
MMP-1	0.86	0.045	0.70 (0.53–0.86)	0.033
VCAM-1/CD106	0.88	0.032	0.68 (0.84–0.51)	0.055
Fas/TNFRSF6	0.83	0.023	0.67 (0.84–0.50)	0.062
IL-17RC	1.39	0.004	0.67 (0.50–0.84)	0.066
LBP	1.26	0.044	0.63 (0.45–0.81)	0.160
Insulin	1.25	0.022	0.61 (0.43–0.79)	0.245
PF4/CXCL4	1.32	0.026	0.60 (0.42–0.78)	0.279
WISP-1/CCN4	1.22	0.031	0.58 (0.40–0.76)	0.387
SPARC	1.21	0.036	0.58 (0.39–0.76)	0.417
Thrombospondin-2	1.26	0.044	0.55 (0.37–0.73)	0.589
Angiostatin	1.25	0.042	0.54 (0.36–0.72)	0.646

The signal intensity of each protein was transformed into a fold-change (HDP/control). *P*<0.05 was considered significant. AUC: The area under the receiver operating characteristic (ROC) curve.

### Functional annotation and clustering analyses

**Fig 3:**
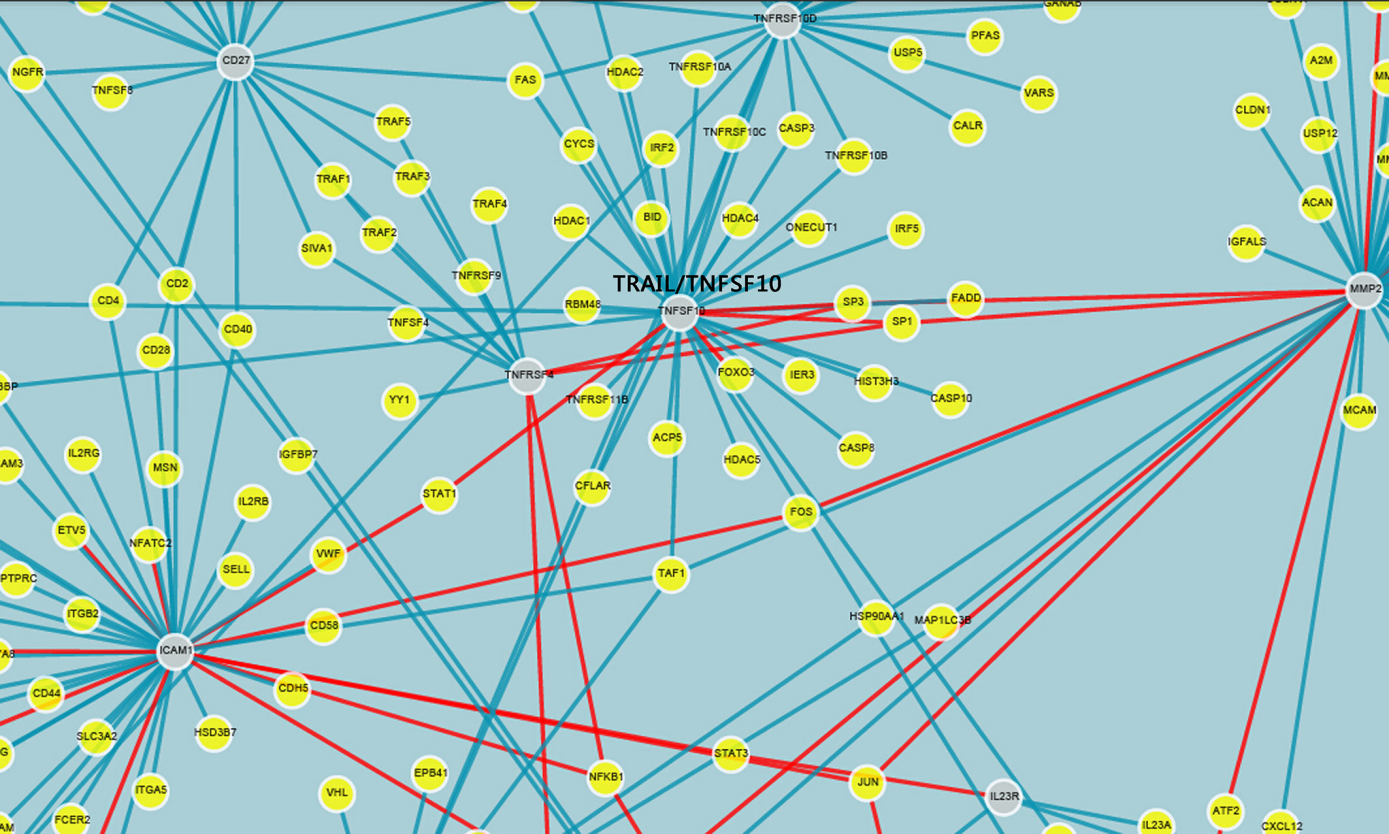
Interaction network of screening proteins in the antibody microarray. TRAIL is the primary connected central protein (hub) interacting with numerous proteins.

The functional annotation and clustering analyses of the 23 proteins were performed using DAVID and the GO database to establish a protein-protein interaction network (Fig 3). The results revealed an enrichment of proteins associated with the extracellular region, cell surface receptor-linked signal transduction, regulation of apoptosis and cell adhesion.

### Validation of TRAIL levels by ELISA

**Fig 4:**
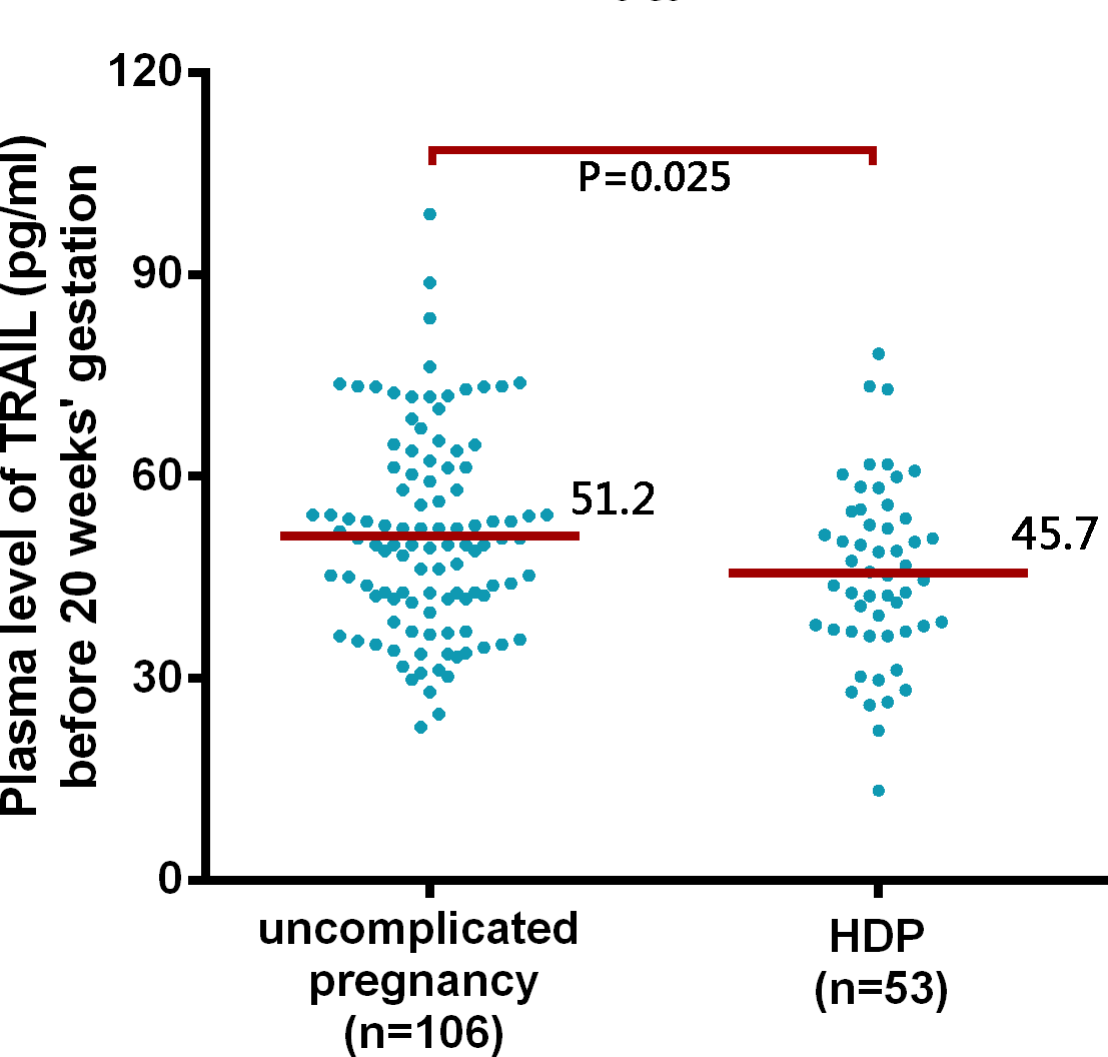
Median plasma level of TRAIL before 20 weeks gestation in women who experienced an uncomplicated pregnancy and women who later developed HDP. Women who later developed HDP had significantly lower TRAIL levels (45.7±13.1 pg/ml, n = 53) before 20 weeks gestation compared to women who experienced an uncomplicated pregnancy (51.2±14.7 pg/ml, n = 106, *P* = 0.025).

Due to the difficulties in obtaining early pregnancy plasma samples from women with HDP, we only validated TRAIL levels because they exhibited the highest fold-change among the down-regulated proteins in the protein microarray, and TRAIL plays an important role in trophoblastic immune privilege and trophoblast invasion during early pregnancy. The results indicated that women who later developed HDP had significantly lower TRAIL levels before 20 weeks gestation compared to women who later experienced an uncomplicated pregnancy (*P* = 0.0.025) (Fig 4).

### Risk factors associated with HDP

To ascertain the risk factors associated with HDP, 53 cases of HDP were matched by age and gestational week to 106 controls, as age and gestational week may be risk factors for HDP. Four significant variables were identified by the univariate analysis (Table 2) and were entered into the multivariable Cox regression analysis. The factors that remained significant in the Cox model included the following: gravidity (OR = 2.02, 95% CI 1.00–4.09), pre-pregnancy BMI (OR = 1.46, 95% CI 1.21–1.76) and TRAIL levels (OR = 0.97, 95% CI 0.94–0.99). The goodness of fit for the Cox model was assessed using the -2Log L statistic. The smaller the -2Log L, the better the model fits the data. The model included TRAIL had a better fit (-2Log L = 76.19) than the model including traditional risk factors alone (-2Log L = 84.77).

**Table 2:** Univariate analysis of candidate prediction factors in the validation set.

Variable	HDP (n = 53) N (%) or mean (sd)	Controls (n = 106) N (%) or mean (sd)	Crude OR (95% CI)	*P*
Family history of hypertension				
YES	17 (32.1%)	13 (12.3%)	3.20 (1.41–7.29)	0.006
Gravidity				
1	30 (56.6%)	75 (70.8%)	1.94 (1.07–3.53)	0.030
≥2	23 (43.4%)	31 (29.2%)		
Pre-pregnancy BMI (kg/m^2^)	21.3 (sd 2.3)	19.4 (sd 2.1)	1.44 (1.21–1.73)	<0.001
TRAIL (pg/ml)	45.7 (sd 13.1)	51.2 (sd 14.7)	0.97 (0.94–0.99)	0.025
Maternal age (years)	29.6 (sd 4.4)	28.5 (sd 2.7)	1.02 (0.92–1.10)	0.089*
Gestation (weeks)	18.1 (sd 3.5)	18.1 (sd 3.5)	1.00 (0.91–1.09)	0.923*

*Women who developed HDP and women who experienced an uncomplicated pregnancy were matched by age and gestational week.

### Efficacy of the predictive model

**Fig 5:**
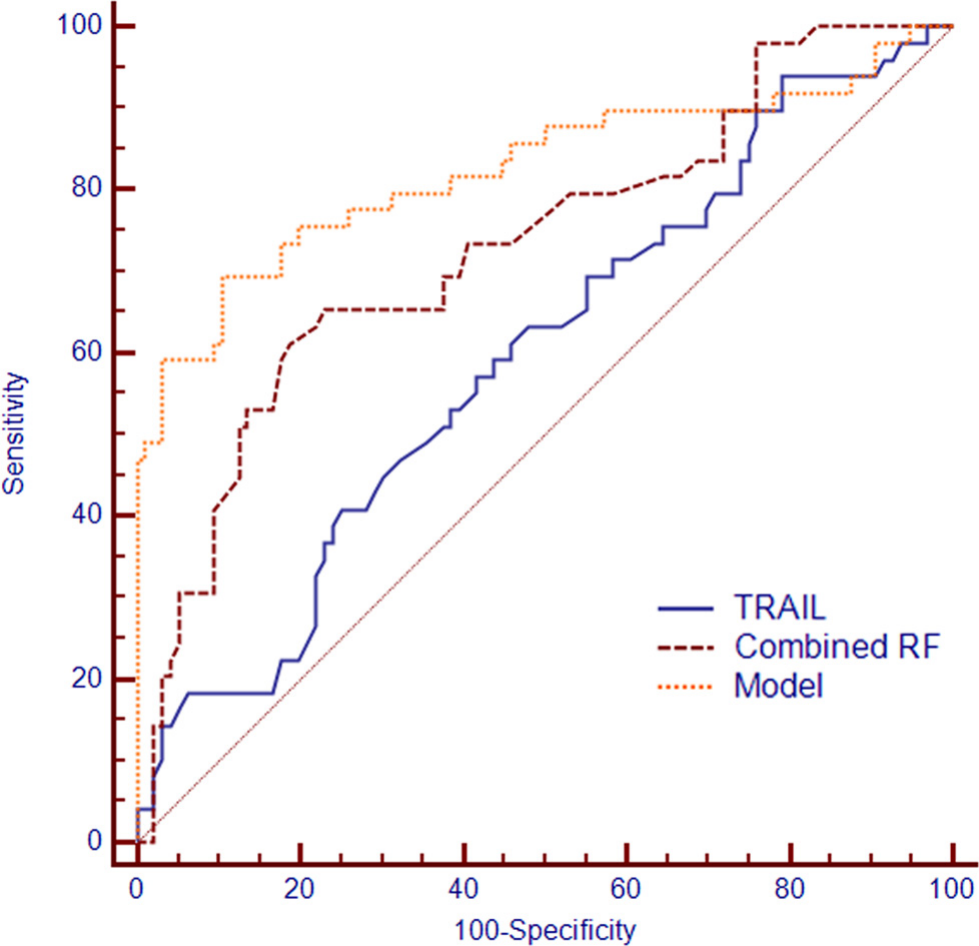
ROC curves of the Cox model for HDP. ROC curves for combined risk factors (RF) alone, TRAIL alone, and the model. TRAIL: AUC 0.59 (95% CI 0.51–0.67); combined risk factors (RF): AUC 0.72 (95% CI 0.65–0.79); model: AUC 0.83 (95% CI 0.75–0.88).

The discriminatory performance of TRAIL and clinical factors associated with HDP were assessed using ROC analysis of TRAIL levels (Fig 5). With TRAIL as a single predictor, the area under the curve (AUC) was 0.59 (95% CI 0.51–0.67) with 60.4% sensitivity and 54.7% specificity based on the optimal cutoff point of 48.9 pg/ml. The AUC increased to 0.83 (95% CI 0.75–0.88) with 70% sensitivity and 90% specificity when clinical factors for HDP were entered into the Cox model based on the optimal cutoff point.

## Discussion

HDP is a complex syndrome with multiple biological changes associated with its etiology. We therefore used antibody microarray technology to determine the key predictive proteins associated with HDP.

To our knowledge, no whole protein expression profiles have been obtained from the early pregnancy plasma of patients with HDP. We identified 11 significantly down-regulated proteins and 12 significantly up-regulated proteins in plasma sampled before 20 weeks gestation from women who later developed HDP using matched controls (Table 1). The 23 identified proteins were allocated into 6 major functional categories: growth factors, adhesion molecules, inflammatory cytokines, chemokines, tumor necrosis factors and matrix metalloproteinases. Functional enrichment revealed that these proteins were predominantly enriched in cell surface receptor-linked signal transduction, regulation of apoptosis and cell adhesion.

TRAIL (also known as Apo2 ligand, Apo2L) interacts with five different receptors in the TNF superfamily. Binding of TRAIL to TRAIL-receptor 1 (TRAIL-R1)/Death receptor 4 (DR4) and TRAIL-R2/DR5 can induce an apoptotic signal through the cytoplasmic death domain (DD). In this interaction, the DD of the TRAIL-receptor complex binds to the DD of Fas-associated death domain protein (FADD), which in turn interacts with the death effector domain (DED) of procaspase-8. Caspase-8 is proteolytically activated, which initiates the caspase cascade, resulting in apoptosis [21]. In contrast, TRAIL-R3/Decoy receptor 1 (DcR1) and TRAIL-R4/DcR2 cannot mediate an apoptotic signal due to complete or partial absence of the cytoplasmic DD; thus, both are described as decoy receptors [22].

TRAIL and its receptors are ubiquitously expressed in various human tissues, principally in immune cells (such as natural killer cells, macrophages, and lymphocytes), endothelial cells, fetal membranes and the placenta [18],[19],[23]. Additionally, TRAIL can induce rapid apoptosis in transformed cells and virus-infected cells. Notably, trophoblast-derived TRAIL induces vascular endothelial cell and vascular smooth muscle cell apoptosis during uterine spiral artery remodeling [17],[24].

TRAIL plays an important role in early pregnancy maintenance. First, TRAIL has been implicated in establishing placental tolerance and immune privilege at the maternal-fetal interface. TRAIL is ubiquitously expressed in the placenta throughout gestation, whereas DcR1 and DcR2 are expressed predominantly in the syncytiotrophoblast, which directly contacts maternal tissues and blood replete with immune cells [19],[25]. Trophoblast-derived TRAIL plays an immune-privileged role to protect the trophoblast from immune rejection by inducing the apoptosis of activated lymphocytes and macrophages of maternal origin [19]. Moreover, the absence of DR and DcR expression in the trophoblast orients TRAIL-induced apoptosis away from the placenta and towards maternal immune cells [26]. TRAIL-expressing NK cells in the decidua are involved in spiral artery remodeling and vascular cell apoptosis [27]. Second, trophoblast-derived TRAIL induces vascular endothelial cell and vascular smooth muscle cell apoptosis and is involved in initiating uterine spiral artery remodeling [17],[24]. This transformation is associated with endothelial cell loss, vascular smooth muscle cell loss, and the replacement of the endothelium by trophoblasts embedded in a trophoblast-secreted fibrinoid matrix. DR4 and DR5 are expressed by spiral artery endothelial cells and smooth muscle cells, and the TRAIL-R-activating antibody produced by trophoblasts induces apoptosis of these cells [17],[24]. Trophoblasts replace endothelial cells and secret a fibrinoid matrix to replace the vascular muscular-elastic layer. Thus, TRAIL protects against immune rejection and promotes spiral artery remodeling.

Although the ability of TRAIL to promote spiral artery remodeling is supported by several lines of evidence, there is a paucity of research describing the association between TRAIL and HDP. Chaemsaithong’s [28] study reported that plasma TRAIL concentrations were significantly lower in patients diagnosed with preeclampsia (22.55 pg/ml) compared to healthy pregnant controls (29.17 pg/ml), suggesting that TRAIL is associated with the pathogenesis of preeclampsia. However, the present study is the first to show that plasma TRAIL levels were decreased before 20 weeks gestation in patients who later developed HDP and were related to HDP (OR = 0.97, *P* = 0.045). In the protein microarray analysis, TRAIL was significantly down-regulated in women who later developed HDP compared to controls (Fig 2; Table 1). The plasma TRAIL concentrations were further validated by ELISA. Before 20 weeks gestation, women who later developed HDP had significantly lower TRAIL levels compared to women who experienced an uncomplicated pregnancy (Fig 4). The decrease in TRAIL before 20 weeks gestation in women with HDP might be associated with failed trophoblastic immune privilege and shallow trophoblast invasion, resulting in reduced placental perfusion.

In the published literature, various risk factors for HDP have been proposed, such as older maternal age, high pre-pregnancy BMI, family history of hypertension and smoking history, but the results are not entirely consistent. In the present study, after adjusting for maternal age and gestational week, factors that remained significant in the multivariable model were gravidity (adjusted OR, aOR, 2.02), pre-pregnancy BMI (aOR 1.46) and TRAIL (aOR 0.97) (Table 3). Our findings are consistent with both domestic and foreign reports [29],[30]. A retrospective study performed by Leeners showed that the risk of developing HDP increased with increasing BMI [29]. Obesity is the principal cause of the metabolic syndrome, which is associated with many pathophysiologic changes, including impaired endothelial function, insulin resistance, and elevated inflammatory markers [31][32]. These physiologic changes could underlie the association between BMI and HDP. Regarding the gravity, HDP is more often developed in older multigravida [33],[34]. In our study, patients are generally old and often have other risk factors for HDP.

**Table 3:** Multivariable Cox regression analysis of candidate predictors in the validation set.

Variable	B	SE	Wald	*P*	Adjusted OR (95% CI)
Gravidity	0.702	0.360	3.797	0.050	2.02 (1.00–4.09)
Pre-pregnancy BMI	0.378	0.097	15.162	<0.001	1.46 (1.21–1.76)
TRAIL	-0.032	0.016	4.012	0.045	0.97 (0.94–0.99)

In the present study, when TRAIL (AUC for TRAIL alone 0.59) was entered into the Cox model as a novel factor, the AUC increased to 0.83 (95% CI 0.75–0.88) with 70% sensitivity, 90% specificity and a high discriminatory performance for HDP (Fig 5). The discriminatory power of our model compares favorably with that of first trimester levels of angiogenic factors. A prospective study by Skråstad et al. reported an AUC of 0.63 for placental growth factor [35]. Abdelaziz et al. observed an AUC of 0.57 for soluble endoglin[7]. However, our model compares unfavorably with the discriminatory power of early pregnancy levels of placental hormones. A longitudinal study by Akolekar et al. reported an AUC of 0.87 for pregnancy-associated plasma protein A and 0.82 for placental protein 13 [36]. It is expected that the discriminatory power of our model will markedly increase when combined with additional biomarkers that were identified in our discovery study.

In conclusion, 23 differentially expressed proteins before 20 weeks gestation might be associated with the pathogenesis of HDP. Notably, our findings validated the decreased plasma TRAIL levels before 20 weeks gestation in patients with HDP, suggesting that TRAIL might contribute to reduced trophoblast invasion into spiral arteries and might serve as a novel predictive, non-invasive marker in maternal plasma for HDP. The data suggest that plasma TRAIL is related to the development of HDP, and plasma TRAIL levels combined with pre-pregnancy BMI and gravidity provided increased discriminatory performance for HDP before 20 weeks gestation.

This study has several strengths. This is a large nested case-control study of 3000 women. The samples were collected before the diagnosis of HDP, from which we found the potential biomarker that might help the early screening of this disorder. Moreover, patients and controls were matched by age, gestational week, and sampling date, avoiding the differences introduced by these factors.

There are some limitations as well. Although our sample pool was large, the HDP sample number before diagnosis was small and limited the precision of our relative risk estimates. Meanwhile, all of the women in our study came from the same province, and this might introduce bias in the results, which should be confirmed by a prospective multicenter clinical study. We reported a model with better performance than other investigators reported. Controls in our study did not include those with abnormal outcomes, which may introduce bias with overestimation of discriminatory power. We only validated TRAIL levels because TRAIL exhibited the highest fold-change among the down-regulated proteins in the protein microarray. In future studies, we will validate the expression of the remaining 22 differentially expressed proteins to further improve the discriminatory performance.

## Supporting Information

S1 File Images of RayBio Human Antibody Microarray. A total of 507 antibodies were placed on the microarray. The complete list of cytokines with their locations and names can be seen at http://www.raybiotech.com/files/manual/Antibody-Array/AAH-BLG.pdf.
(DOCX)

S2 File Plasma concentrations of TRAIL before 20 weeks gestation in women who experienced an uncomplicated pregnancy and women who later developed HDP were measured by ELISA. Assays were performed in accordance with the manufacturer's instructions (http://www.rndsystems.com/pdf/dtrl00.pdf).
(XLSX)
