# Supplementary material for: TRAIL Is Decreased Before 20 Weeks Gestation in Women with Hypertensive Disorders of Pregnancy
Source: PLoS One. 2015 Jun 1;10(6):e0128425. doi: 10.1371/journal.pone.0128425 (PMC4451764; doi:10.1371/journal.pone.0128425)

**HDP**

Sample 1 Sample 2 Sample 3 Sample 4 Sample 5


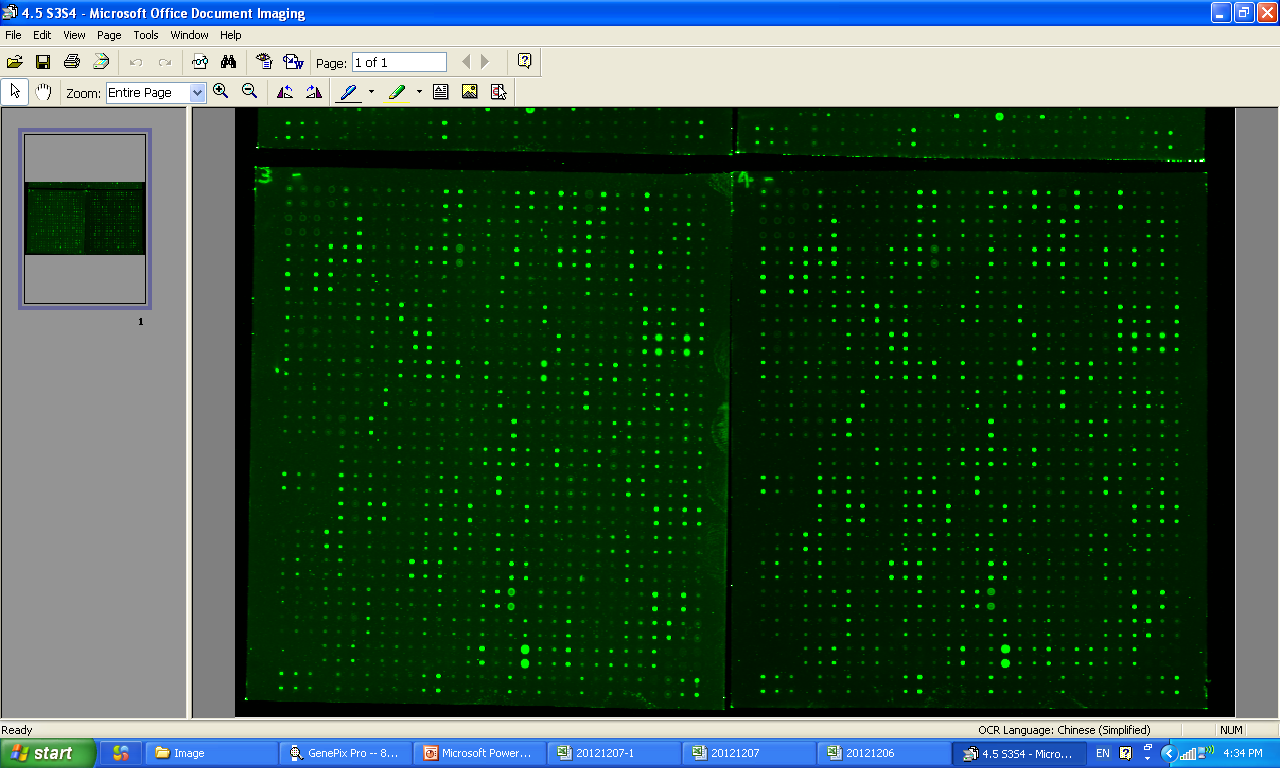

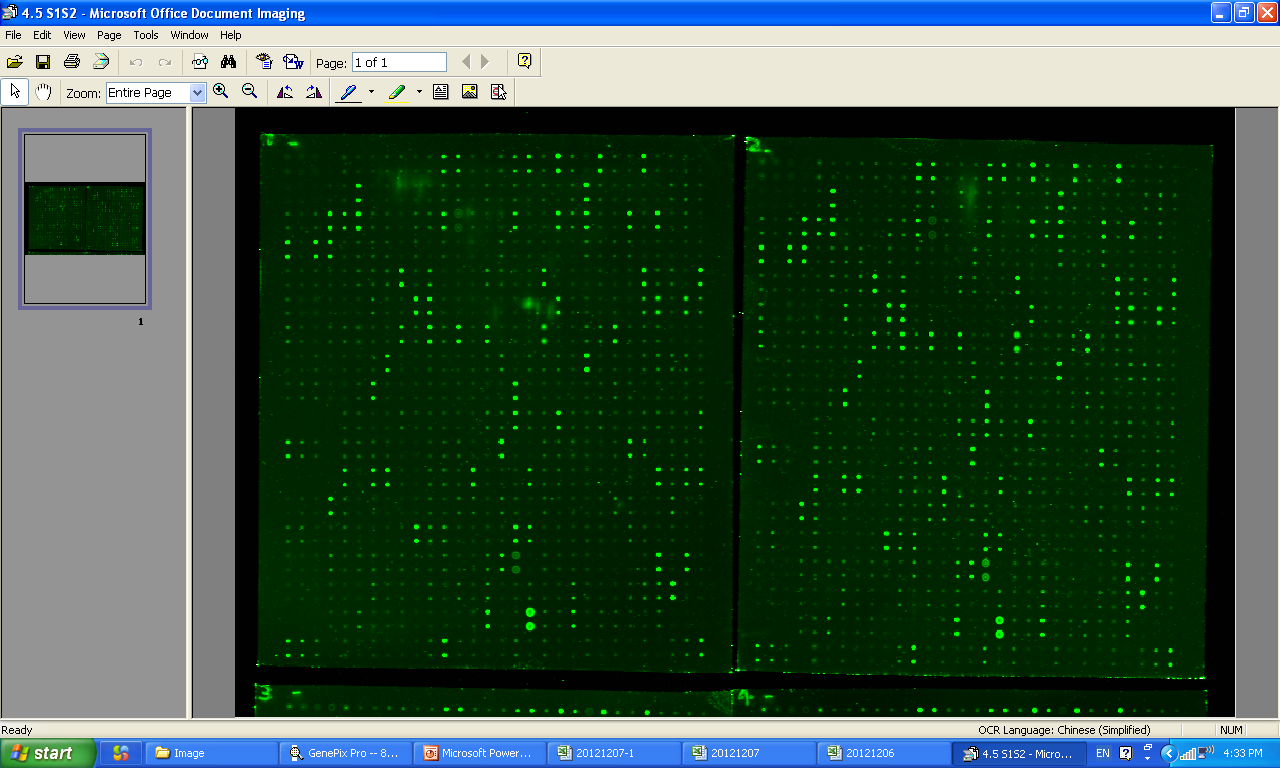

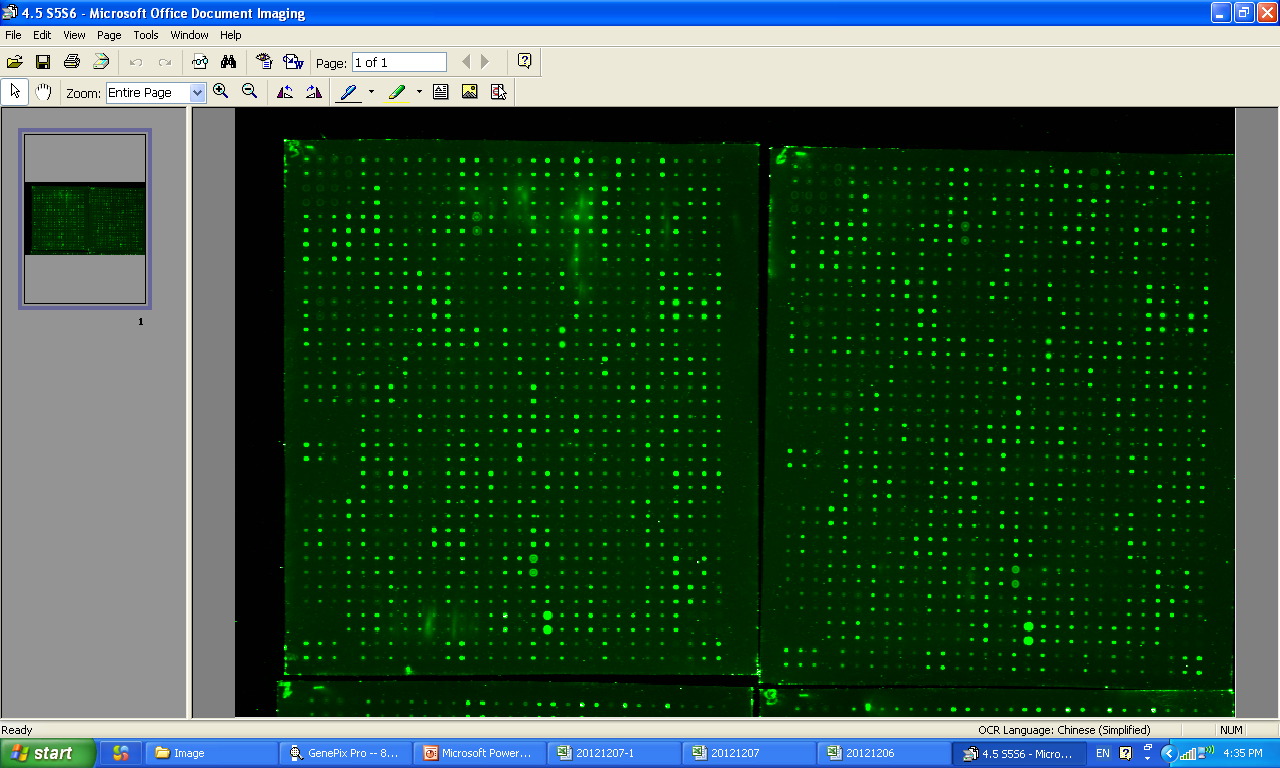

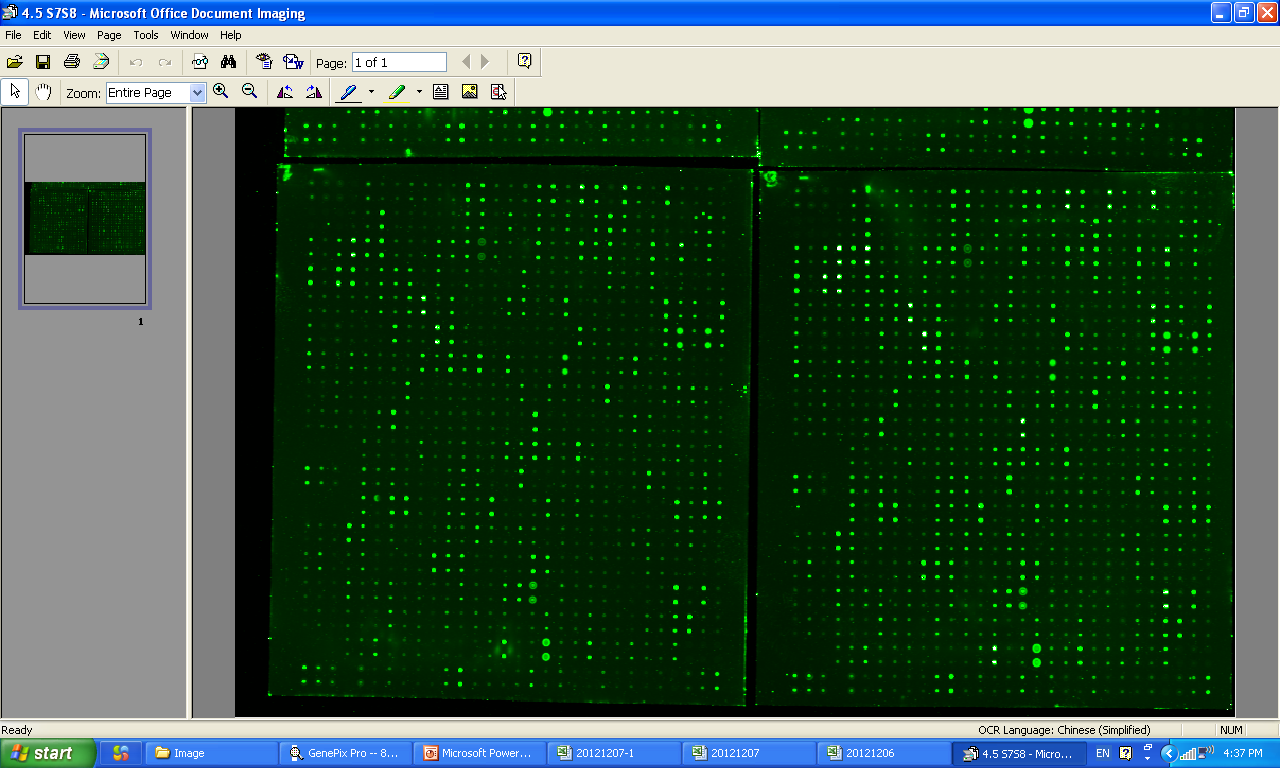

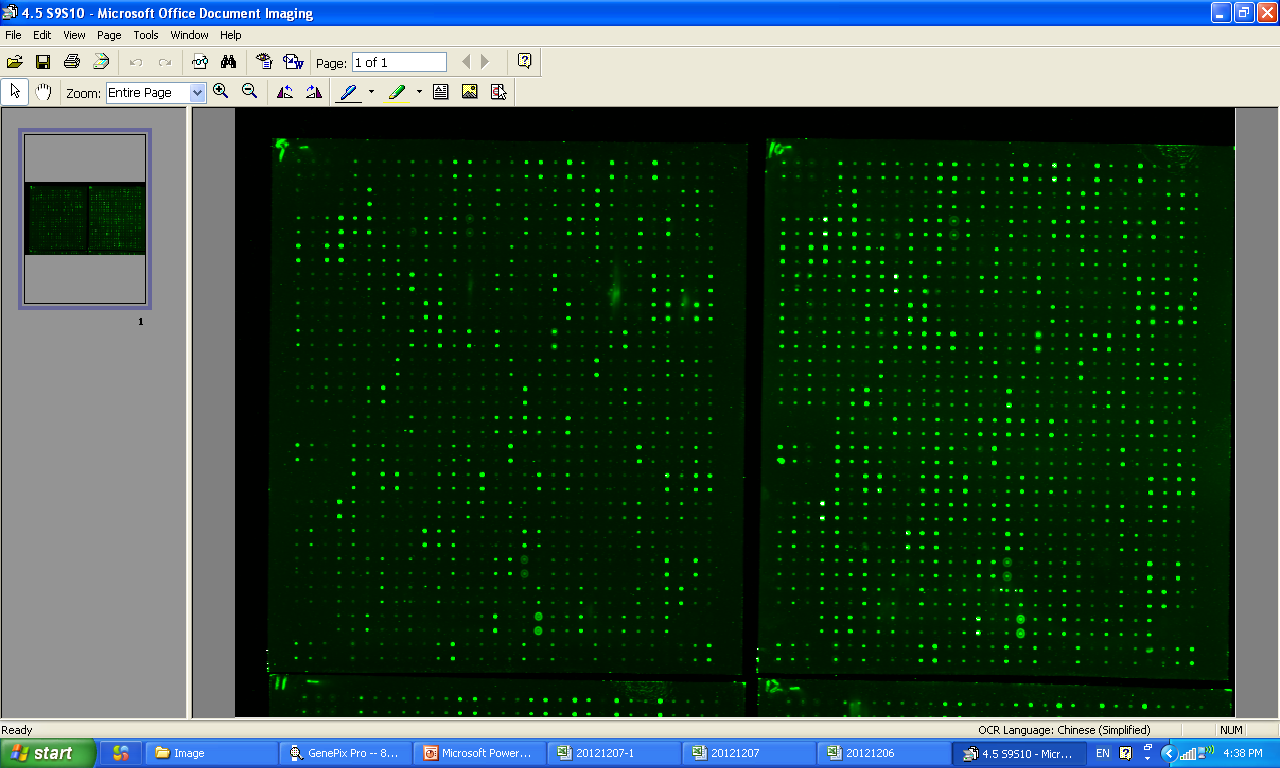


Sample 6 Sample7 Sample 8 Sample 9 Sample 10


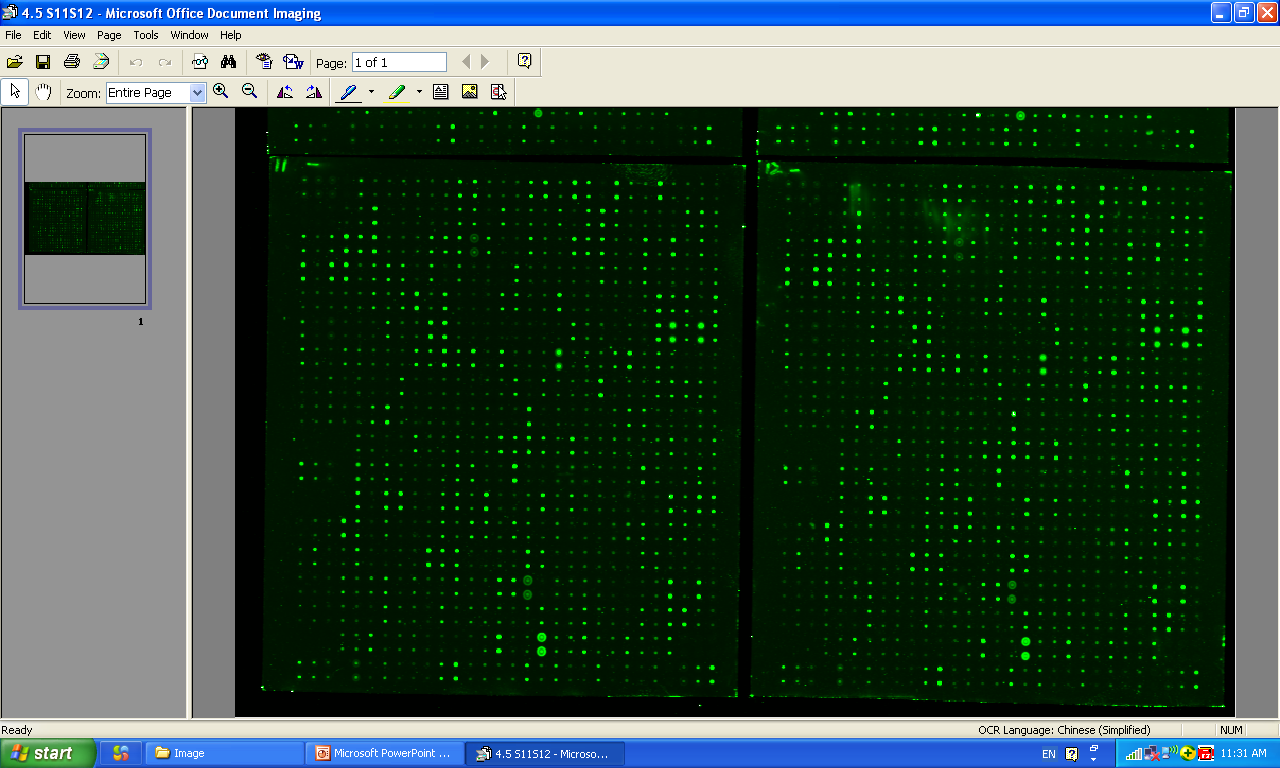

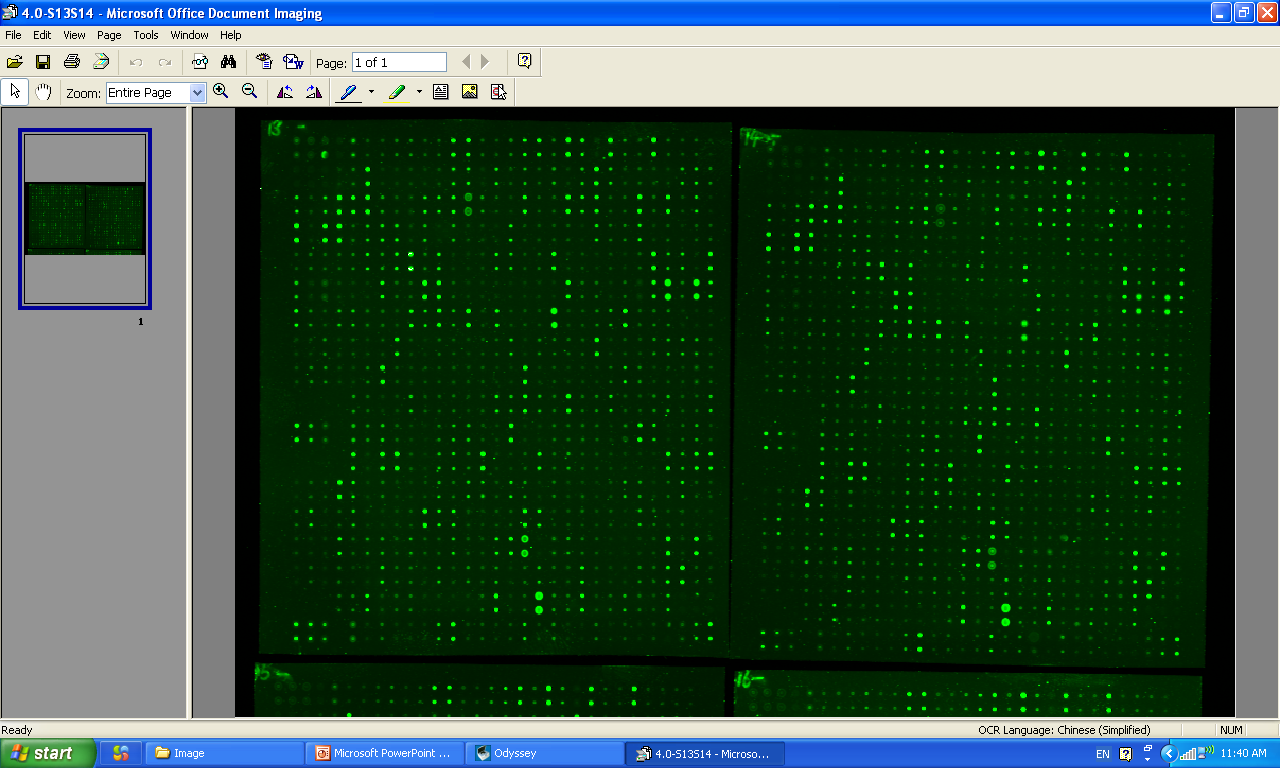

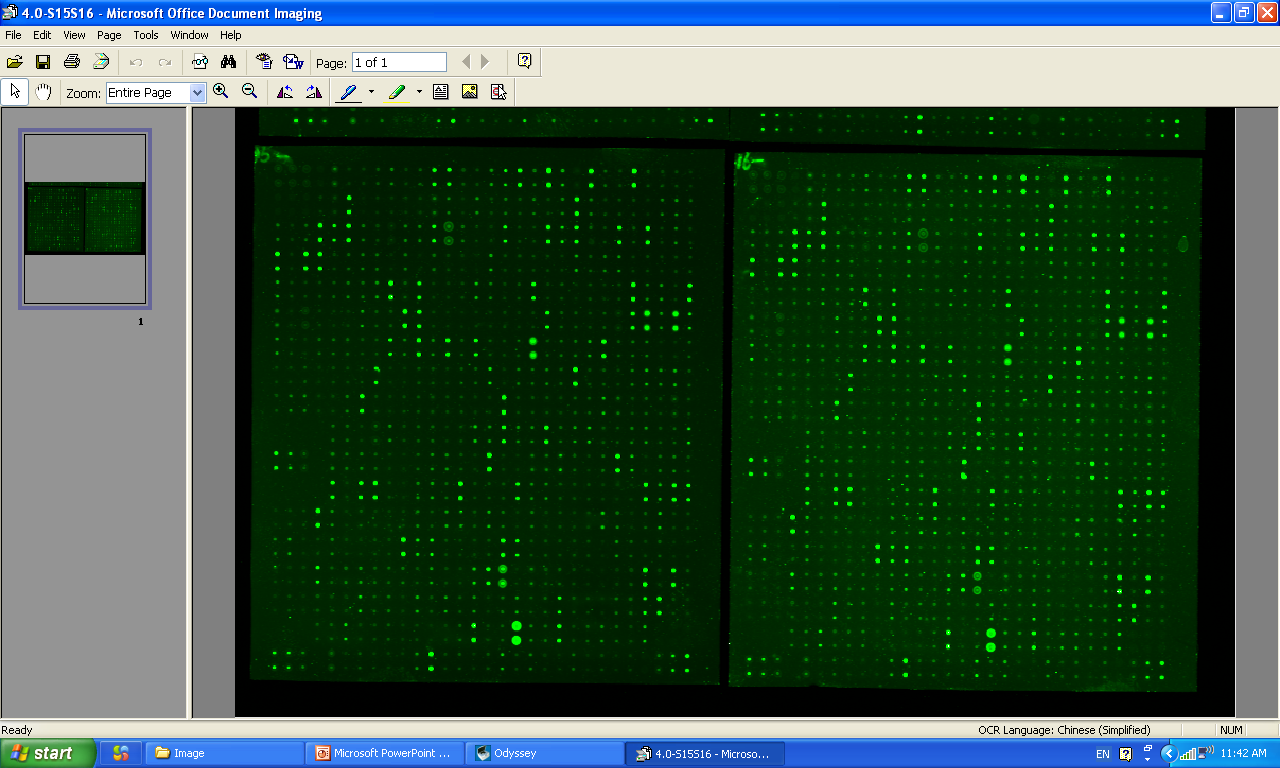

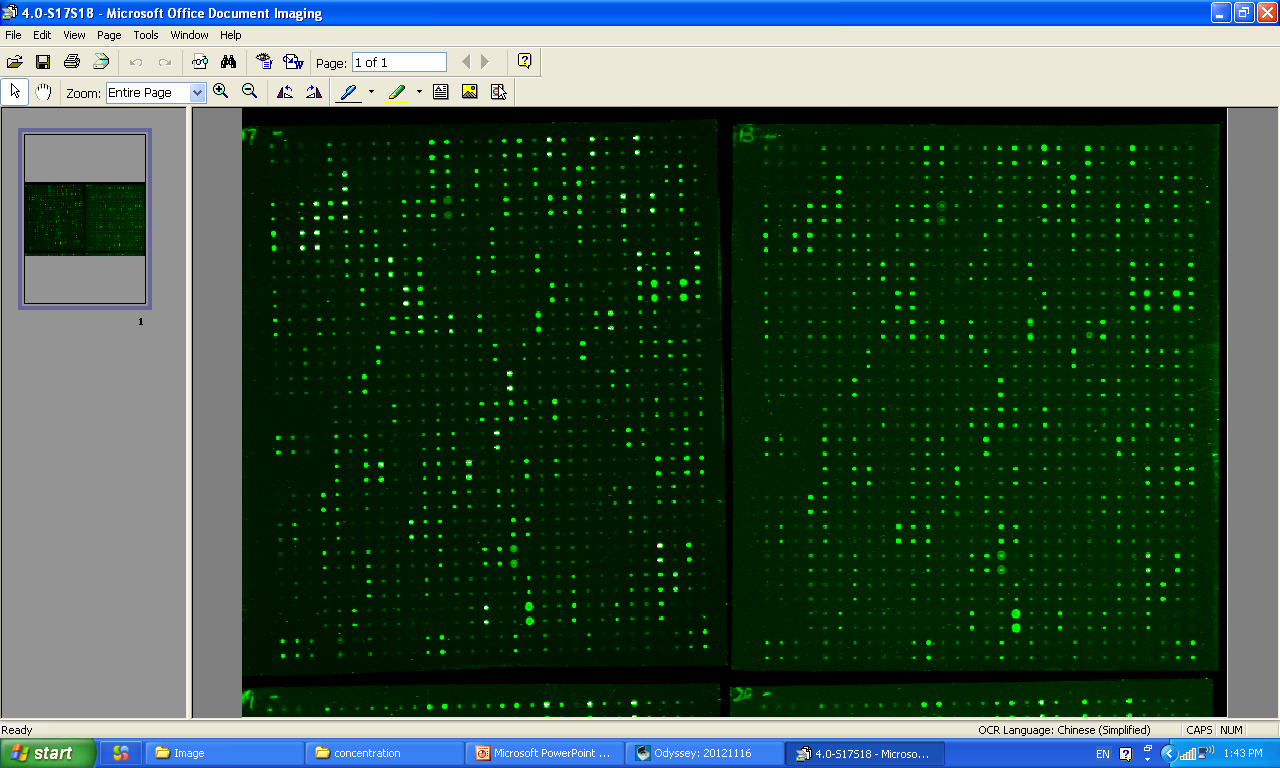

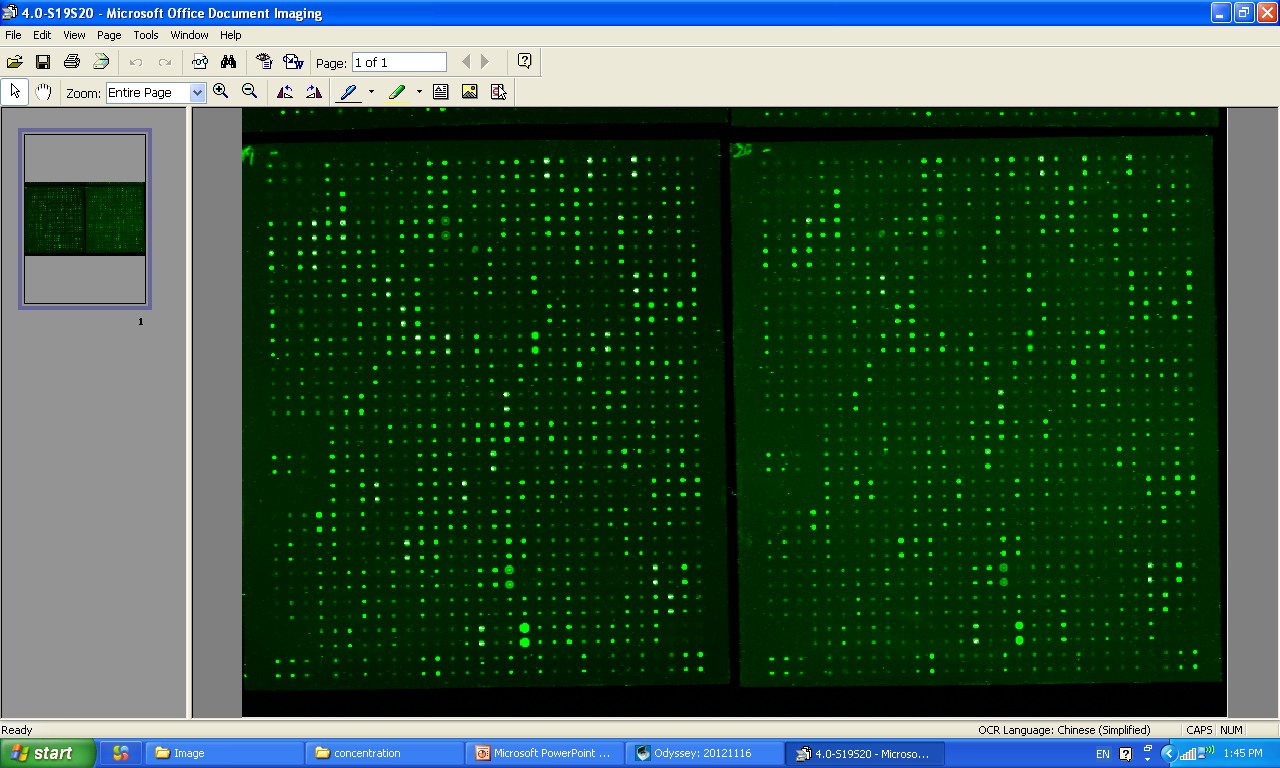


**Healthy pregnancy**

Sample 1 Sample 2 Sample 3 Sample 4 Sample 5


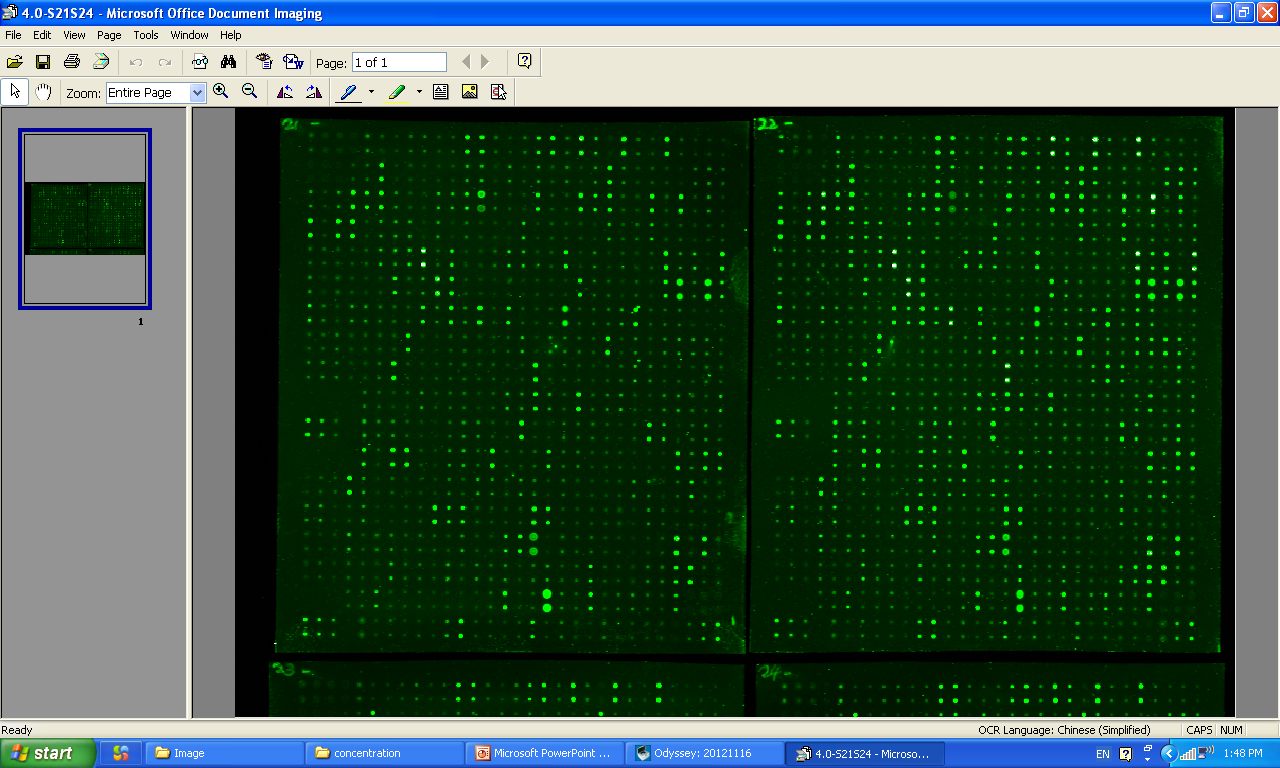

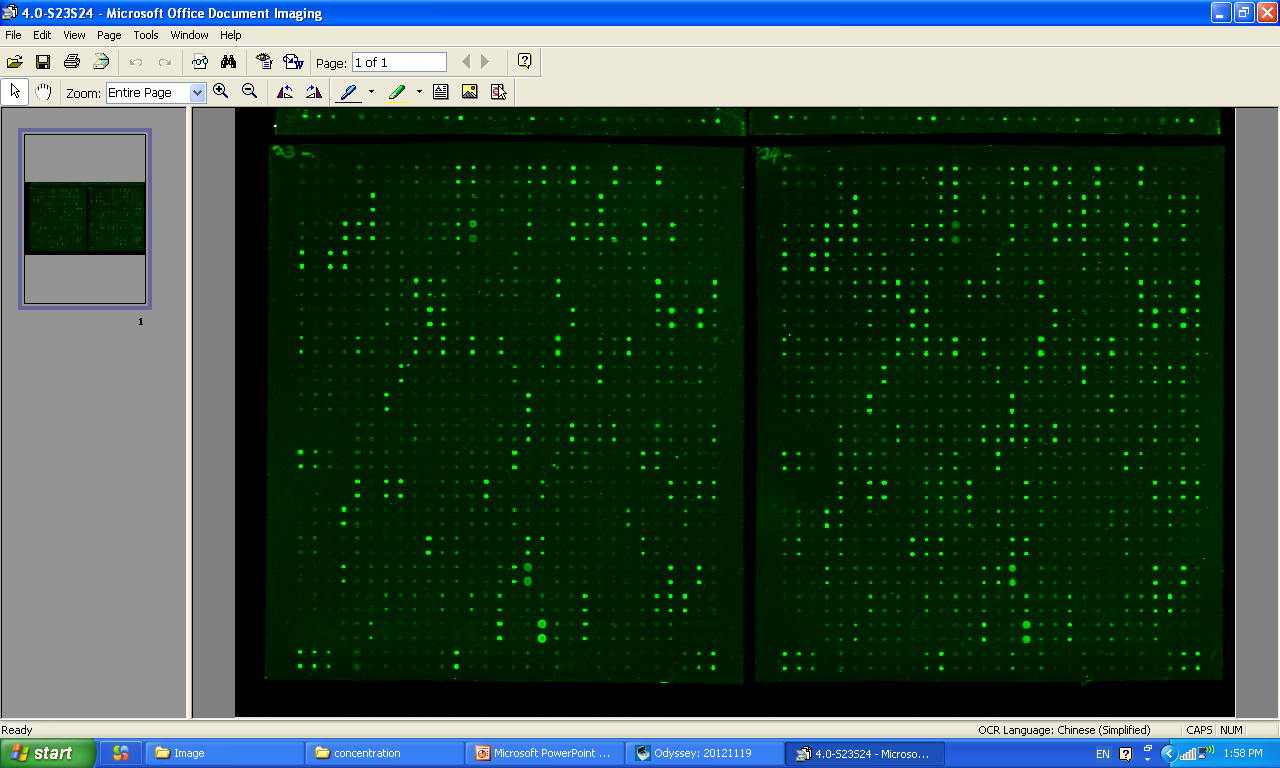

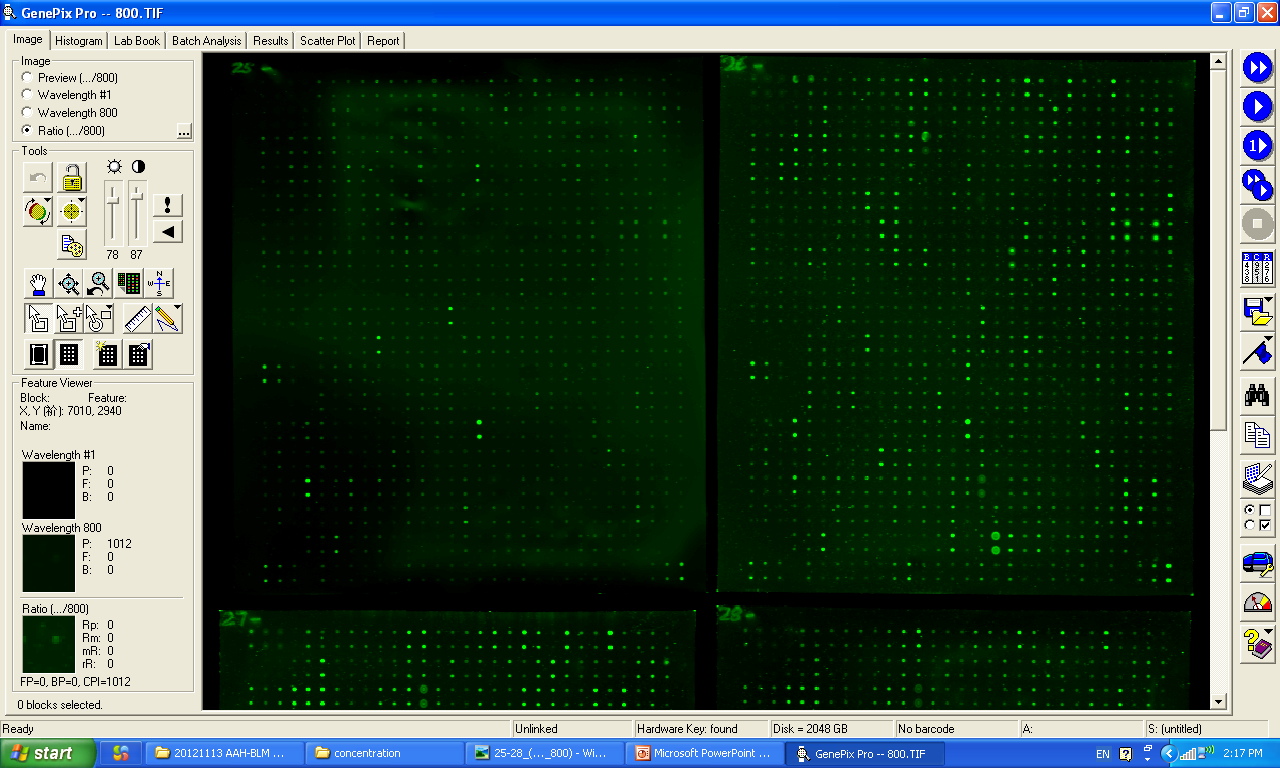

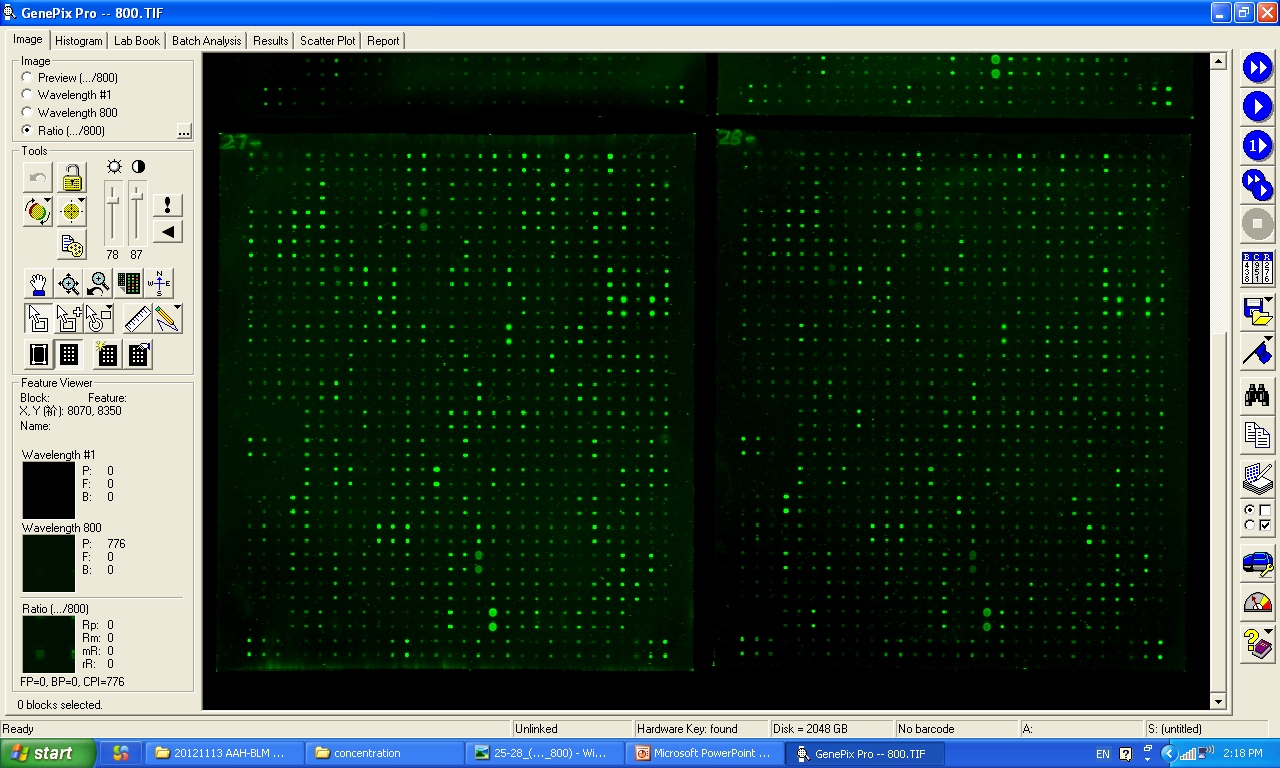

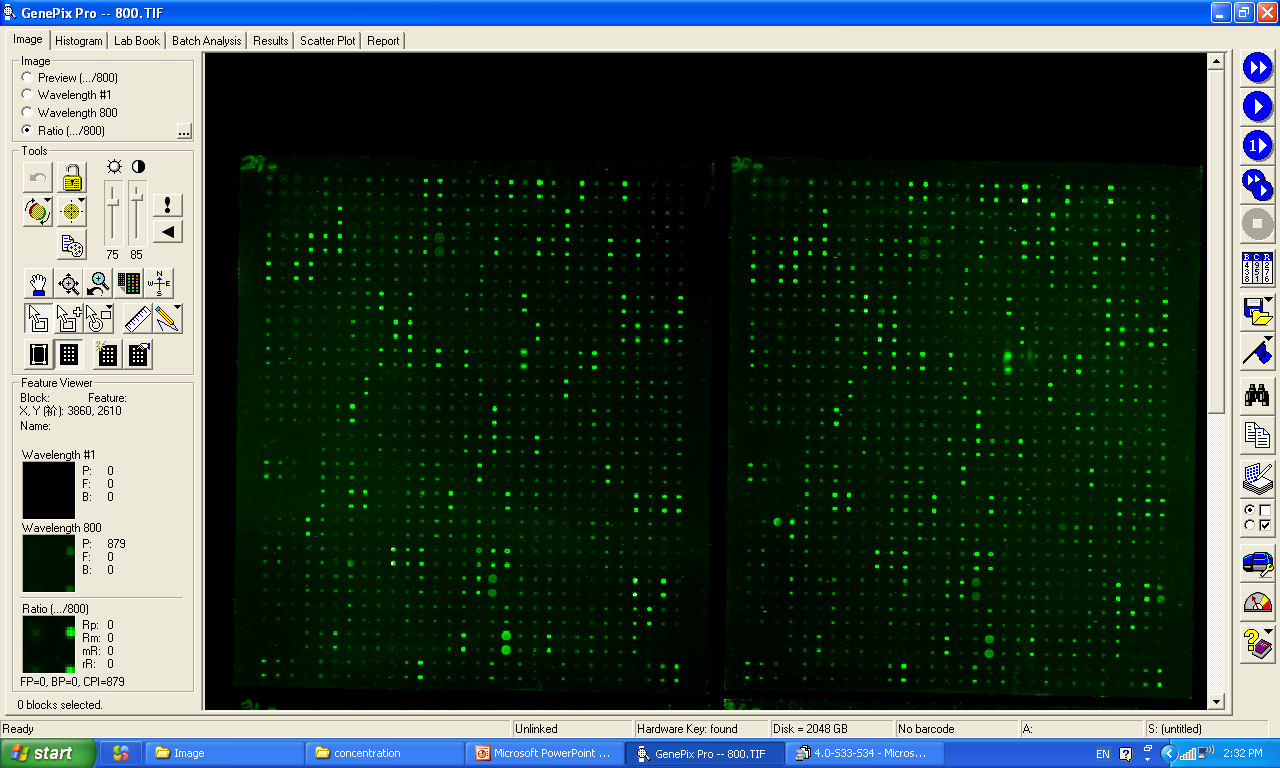


Sample 6 Sample 7 Sample 8 Sample 9 Sample 10


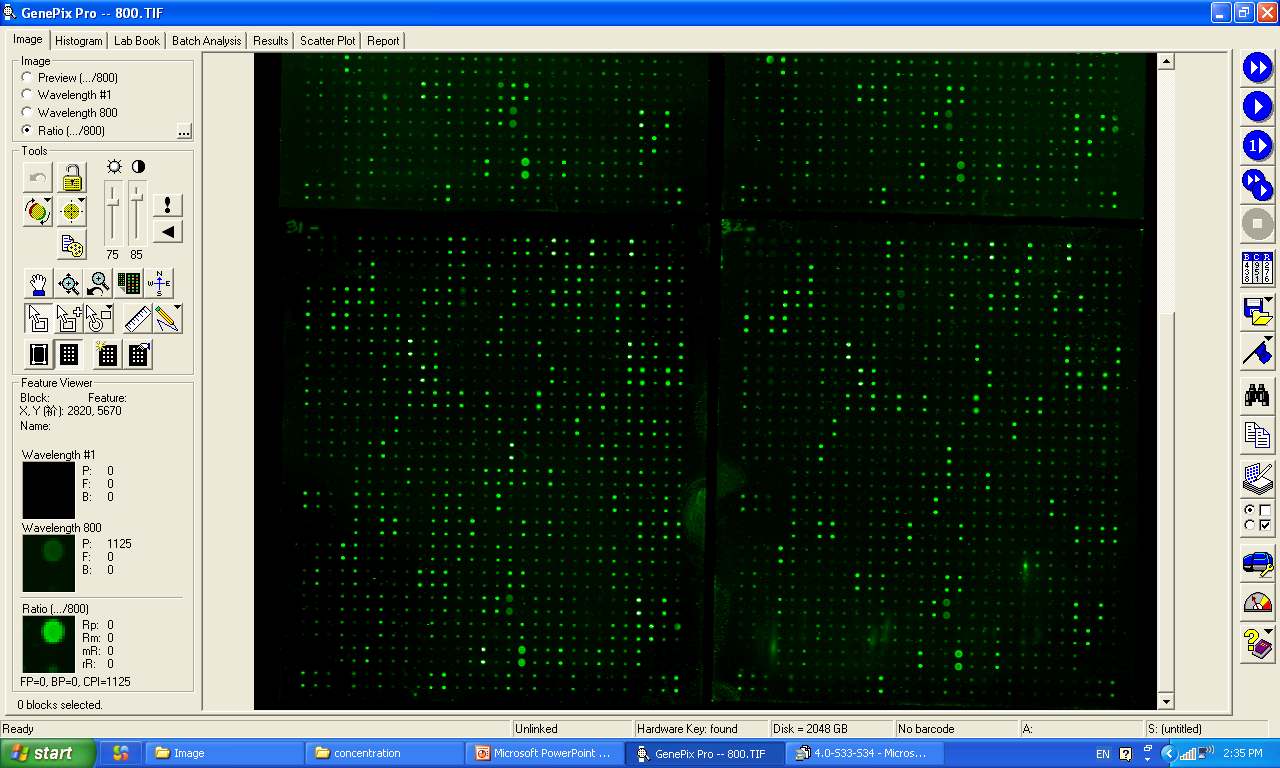

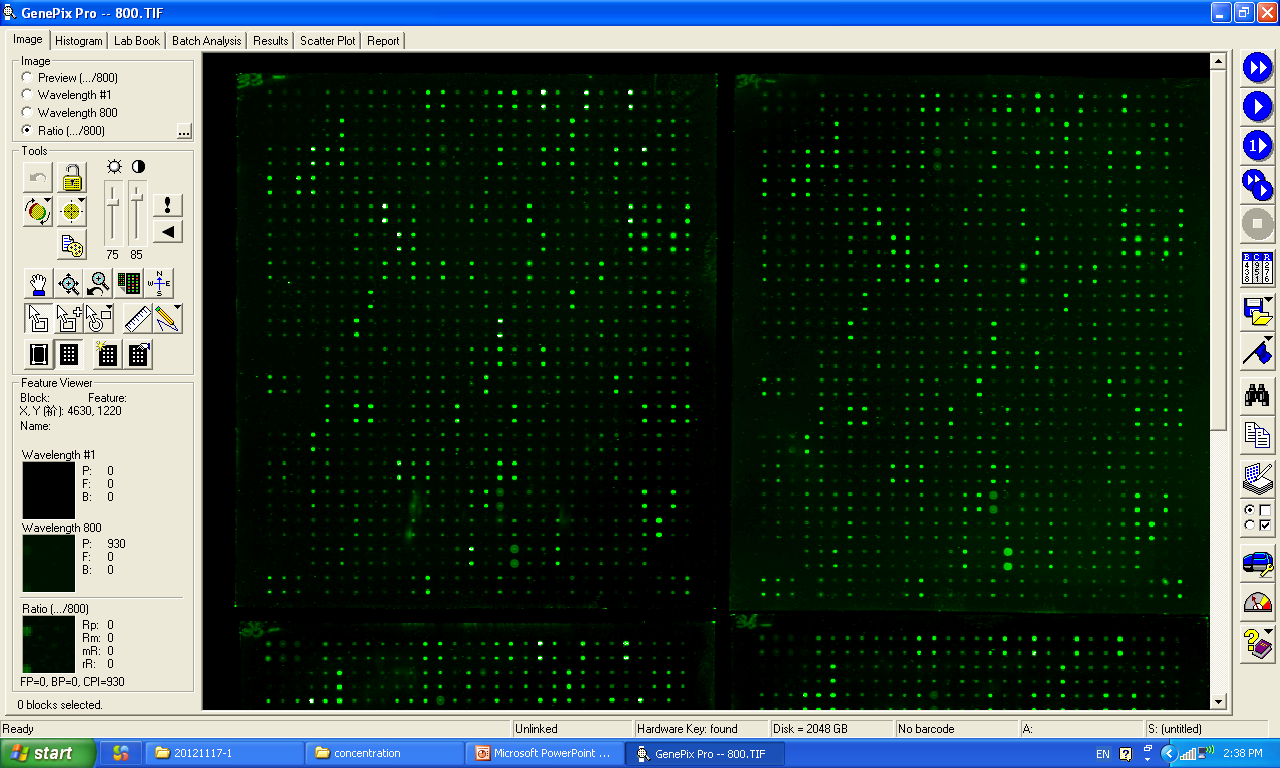

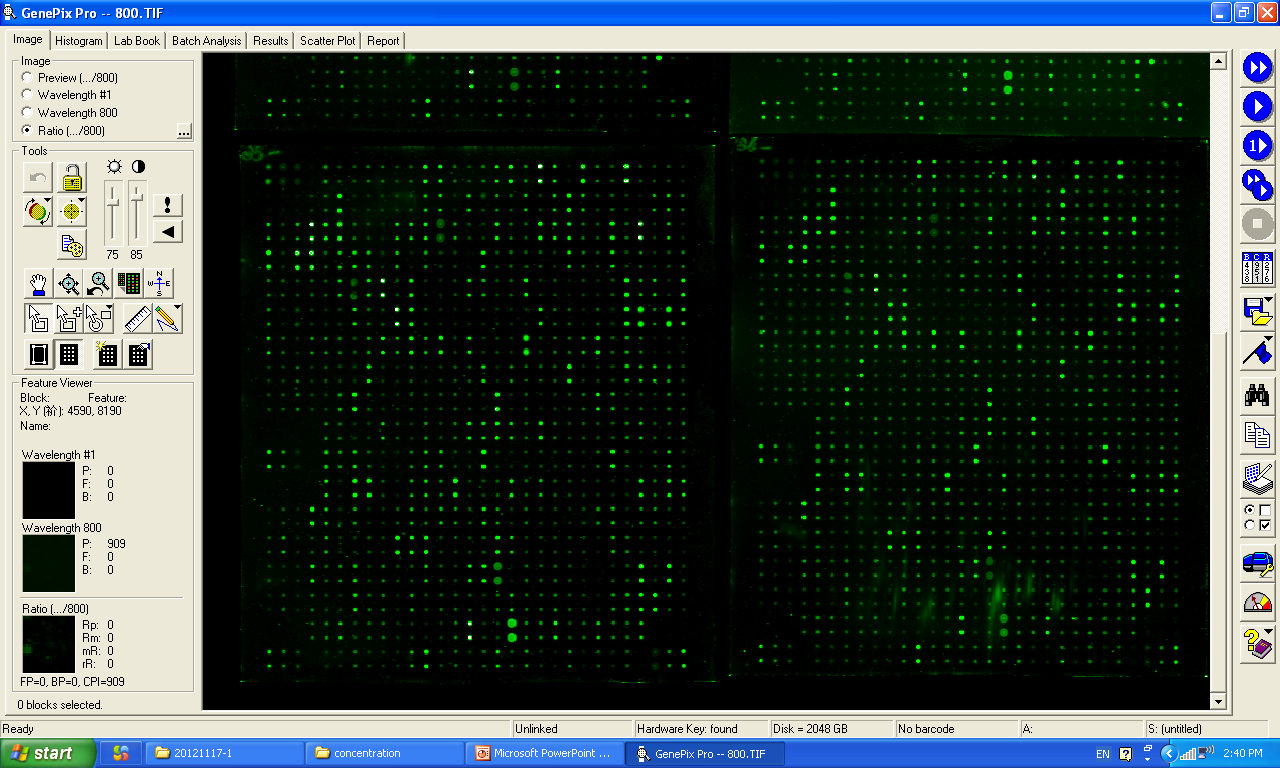

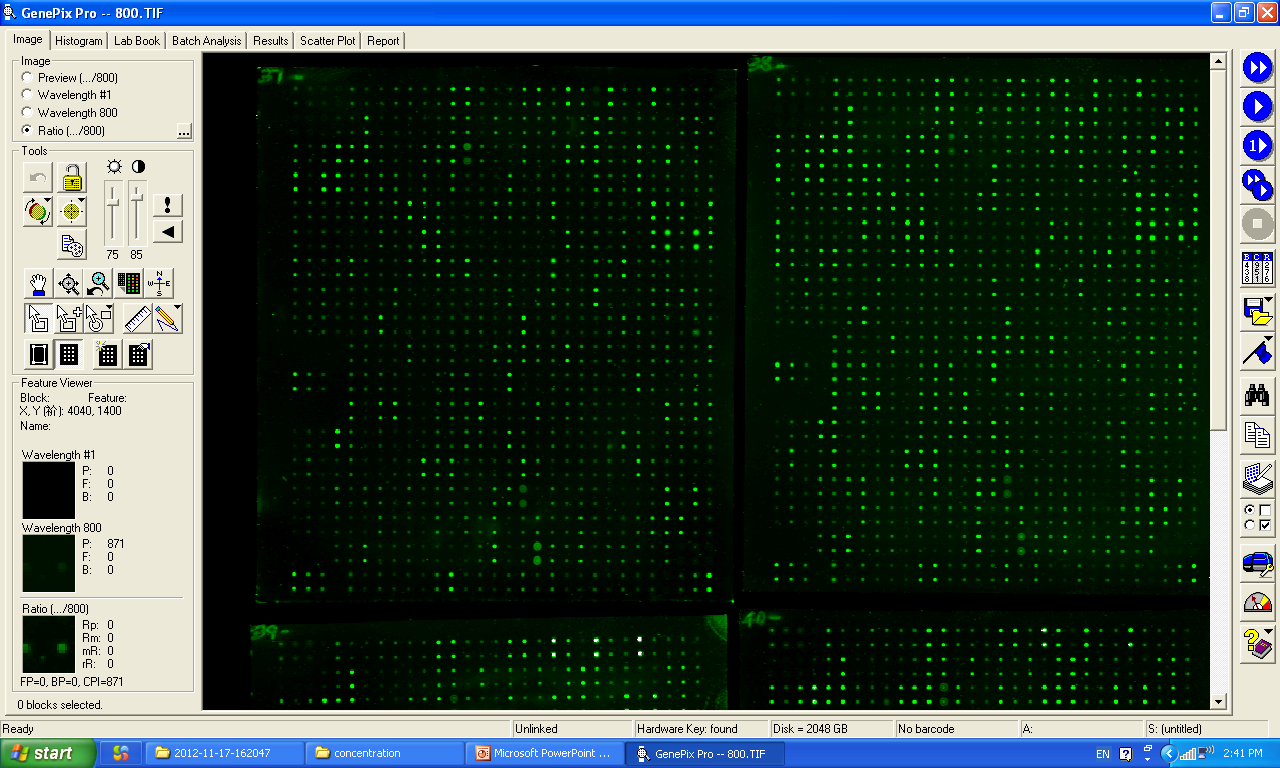

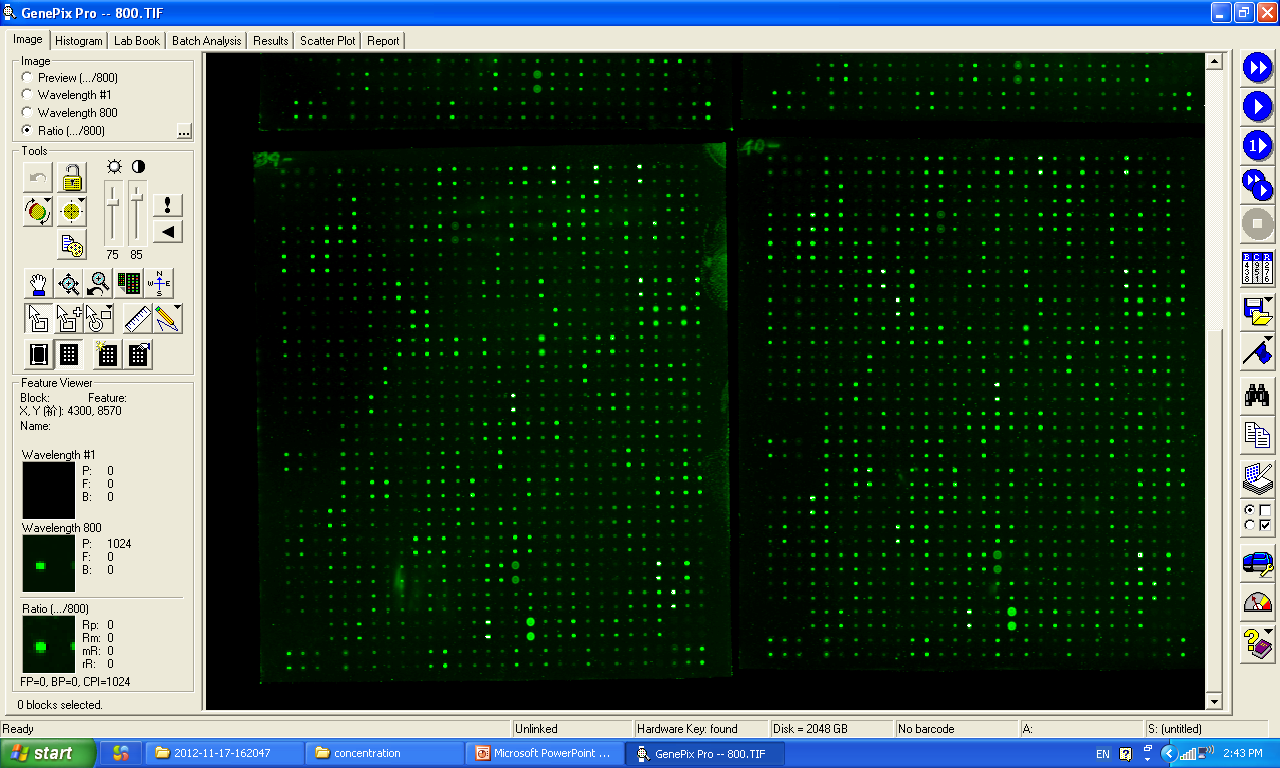

Supplement: S1 File — A total of 507 antibodies were placed on the microarray. The complete list of cytokines with their locations and names can be seen at http://www.raybiotech.com/files/manual/Antibody-Array/AAH-BLG.pdf. (DOCX) [file pone.0128425.s001.docx]
